# Resident-led Implementation of a Standardized Handoff System to Facilitate Transfer of Postoperative Neurosurgical Patients to the ICU

**DOI:** 10.7759/cureus.461

**Published:** 2016-01-18

**Authors:** Harjus S Birk, Seunggu J Han, John D Rolston, Nathan C Rowland, Catherine Lau, Philip V Theodosopoulos, Michael W. McDermott

**Affiliations:** 1 Department of Neurological Surgery, University of California, San Francisco; 2 Research Fellow, Howard Hughes Medical Institute; 3 Department of Neurological Surgery, University of Toronto

**Keywords:** perioperative care, surgical handoff, transfer of care, interdisciplinary communication

## Abstract

Transitions in care are pivotal moments for patient safety. Although many strategies have been suggested for handoff improvement in the healthcare realm, little focus has been placed on patient safety during the transition from the operative to the postoperative setting. Many surgical trainees have received limited instruction, if any, on how to conduct comprehensive handoffs that ensure the safe transition of care and optimize continuity of care. Therefore, structured transfers of patient care can be invaluable. Here, we describe the implementation of a standardized handoff system developed by residents in an academic neurosurgery department to communicate key perioperative data via both electronic documentation and in-person discussion as a means of reinforcement. Our results are part of a comprehensive effort to strengthen the culture of safety surrounding the care and treatment of neurosurgical patients at our institution.

## Introduction

Recent attention has been given to quality initiatives that address inpatient neurosurgical care; however, patient safety and quality of care issues specific to the immediate perioperative period are less well described [1-2]. Since this setting involves a major transition of care with high potential for a breakdown in communication secondary to the highly stressful environment, it is essential to maintain coordinated communication strategies that can be implemented in order to ensure patient safety and promote continuity of care. Structured transfers of patient care are often referred to as handoffs, which Cogen and Hilligoss defined as “the exchange between health professionals of information about a patient accompanying either a transfer of control over, or of responsibility, for the patient” [3]. 

These transfers of care have been developed for a number of other surgical subspecialties and have been shown to reduce discrepancies in interdisciplinary communication between providers [4-5]. For example, in 2015, Dixon, et al. analyzed the implementation of a transfer of care system for cardiac surgical patients from the OR to the ICU. A formalized handoff process from resident to registered nurse included a standardized handoff procedure with a checklist; data collected from 60 observed handoff encounters between physicians and nurses revealed improved key data transmission that ultimately resulted in reduced median time until ICU monitor transfer and measurement of the first cardiac index [6]. Another study among pediatric cardiac patients evaluating the use of a Formula One-based handoff led to the conclusion that restricting handoffs to simply information documentation without social interaction was not efficacious [7].

Given the complexity of care provided in the preoperative setting for neurosurgical patients, we developed a systematic handoff approach to facilitate the transfer of care for patients transitioning from the operating room to the postoperative setting. In this study, we took into consideration the lessons learned from the aforementioned studies, and employed a bimodal and systematic strategy in pairing the use of an electronic form documenting perioperative data in a timely manner with a face-to-face handoff system between physicians and residents to facilitate patient transfers to the neurosurgical intensive care unit (ICU). 

The passage of The Accreditation Council for Graduate Medical Education (ACGME) work-hour limitation rules for resident physicians likely results in more handoffs that may occur, putting patient safety in jeopardy if a systematic handoff system is not followed. Neurosurgery programs are more vulnerable to the handoff situation due to a smaller ratio of residents to a number of patients on the service, and therefore, our specialty holds a need for a comprehensive handoff system that facilities patient safety, especially among interdisciplinary communication.  In 2012, Babu, et al. sent a validated 20-question online survey to 795 neurosurgical residents of various neurosurgery programs throughout the United States to assess familiarity and training with handoffs and communication strategies, and 449 surveys were completed [8]. Ninety percent of the residents reported that handoffs most often occur in private, quiet areas that may not be representative of the hectic nature when transitioning from the operative to postoperative setting. Sixty-three percent of residents surveyed had not received instruction in what constitutes an effective handoff, and 24% reported moderate-to-high variability among residents in handoff quality to other healthcare professionals.     

Studies have also shown that effective handoff communication strategies should consist not only of passive processes, such as electronic documentation, but also face-to-face communication that enables conversation during which questions can be posed and clarification can be obtained [9-11]. In order to overcome this hurdle and incorporate an active component, our handoff process for relaying perioperative data consists of both electronic documentation as well as face-to-face discussion with a registered nurse, enabling a bimodal and systematic strategy.

This paper describes the characteristics of our handoff system to ease the transition of neurosurgical patients to the ICU in the immediate postoperative period, consisting primarily of a brief postoperative note and an in-person encounter with another healthcare professional. This system was led by residents at our institution and was well-received with user satisfaction. The purpose of this paper is to describe our experience in both developing and implementing a system for transfer of patient care to relay perioperative data about neurosurgical patients to other healthcare professionals, while minimizing distractions common in the hectic surgical environment. Although the challenge of the 80-hour resident workweek may result in more interdisciplinary handoffs, the goal of our standard interdisciplinary postoperative handoff is to ensure that neither patient care nor safety is jeopardized and, ultimately, work efficiency is maintained.

## Materials and methods

The following initiative was developed and implemented by neurosurgical residents at the University of California, San Francisco (UCSF), in 2012.

A note template entitled ‘Brief Op Note’, modified from a prior version, was created using the Epic^®^ digital medical record system (Epic Systems, Verona, WI, USA). The template was inserted into patient transfer-of-care ‘Navigators’ that the system automatically highlights based on an algorithm that tracks patient movement throughout the UCSF surgical realm. Additionally, the note could be initiated manually without the use of a Navigator by opening a progress note and entering a pre-defined “smartphrase.” The note contained the following fields: surgeon, date of operation, preoperative diagnosis code, procedure, implants, and specimens. The surgeon and procedure fields contained modifiers describing role (e.g., primary vs resident - assisting) and anesthesia type (e.g., general), respectively. 

These fields were pre-populated based on circulating nurse input throughout the duration of the operation. Additional fields prompted for user input were a postoperative diagnosis, the exact procedure performed, pertinent intraoperative findings (such as frozen pathology results and neurophysiological monitoring results), fluids, estimated blood loss, wound classification, incisional closure type, drains, and expected disposition and plan. While the surgical procedure was automatically populated from the case bookings Current Procedural Terminology (CPT) code, the custom note required an additional free text entry of the procedure, since this is often more informative than the CPT code. Fields requiring user input could be advanced using keyboard shortcuts and were prompted for completion if the note was attempted to be prematurely signed without completion of all required fields. All fields requiring user input allowed free text, except wound classification and incisional closure classification, both of which contained predefined items in dropdown format. 

Neurosurgical residents were asked to complete the notes after transferring the patient to the postoperative setting in a timely manner within 30 minutes of leaving the OR or PACU. This time period was chosen because 30 minutes was viewed as ample time to complete the notes following surgery. An additional requirement was to perform a face-to-face handoff with the assigned postoperative registered nurse (RN) as of means to repeat the elements of the brief postoperative note. Finally, a dropdown box at the end of the brief operative note was inserted to indicate whether the face-to-face handoff had been completed by the resident. Nurses were informed that a new brief operative note would be available (completed by the same resident performing the face-to-face handoff) to assist in the transfer of care for each patient.

To measure neuro ICU RN and neurosurgical resident attitudes towards this new brief operative note, a survey instrument was created. Separate surveys for RNs and residents were administered, consisting of 14 and 18 questions, respectively (Tables 1-2). RNs and residents who agreed to participate were explained the nature and the objectives of the study, and informed consent was formally obtained. 

**Table 1:** Survey Questions for ICU RNs

Survey Questions for ICU RNs	
What is your primary source for information regarding the exact procedure performed?	
How often do you feel confident in your understanding of the exact procedure performed?	
How often do you feel confident about your understanding of the patient’s postoperative pain level?	
How often do you need to contact the on-call resident to clarify the exact procedure performed?	
How often do you need to contact the on-call resident to clarify questions regarding the patient’s postoperative pain level?	
How often do you see the exact procedure documented in the chart immediately following the procedure (i.e., less than 30 min)?	
How often do you have to look elsewhere other than in EPIC to find the exact procedure performed?	
How often does the lack of communication/documentation of the exact procedure performed negatively impact care of the postoperative patient?	
How important is immediate documentation of the exact procedure performed?	
How important is immediate documentation of fluids administered?	
How important is immediate documentation of estimated blood loss?	
How important is immediate documentation of preliminary pathology?	
How important is immediate documentation of postoperative disposition?	
Brief operative notes are a useful source of information in my clinical practice (Agree/Disagree)	

**Table 2:** Survey Questions for Neurosurgery Residents

Survey Questions for Neurosurgery Residents
What is your current postgraduate year?
How often do you see the exact postoperative procedure performed immediately following the OR procedure (less than 30 min)?
On average, how often are you called to provide care for an immediate postoperative patient (within 4 hours) while on call?
How often do you have to look elsewhere other than EPIC to find the exact procedure performed?
How often does the lack of communication/documentation of the exact procedure performed negatively impact care of the postoperative patient?
On average, how soon after the conclusion of the case is the exact procedure verbally communicated to you?
When would you like to know the exact procedure performed?
How important is immediate documentation of the exact procedure performed?
How important is immediate documentation of fluids administered?
How important is immediate documentation of estimated blood loss?
How important is immediate documentation of preliminary pathology?
How important is immediate documentation of postoperative disposition?
How often do you receive verbal feedback from others regarding the lack of information about a recently completed surgery (including physicians, nurses and other healthcare workers)?
On average, what percentage of cases that you are involved in do you complete brief operative notes?
Brief operative notes are a useful source of information in my clinical practice. (Agree/Disagree)
Brief operative notes take too much time to complete. (Agree/Disagree)
Brief operative notes provide helpful clinical documentation to my colleagues. (Agree/Disagree)
Brief operative notes improve the safety of care provided to postoperative patients. (Agree/Disagree)

The surveys were designed using the Qualtrics^®^ web-based survey creation platform (Qualtrics, Provo, UT, USA). The RN survey consisted of 14 questions and the resident survey consisted of 18 questions. Response data from each survey question were aggregated in percentiles and accompanied by descriptive statistics (i.e., min, max, mean, variance, and standard deviation). RNs and residents received an email with a hyperlink to the web-based survey.

## Results

**Figure 1:**
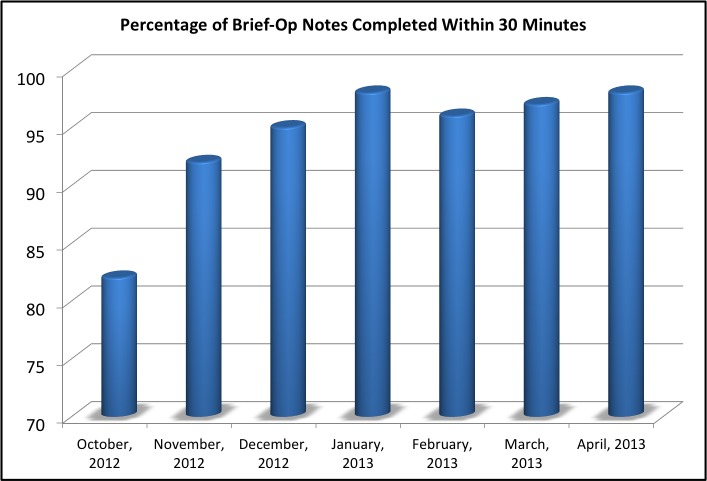
Percentage of Brief-Op Notes Completed Within 30 minutes This diagram shows that from project initiation to the end of the study period, the brief operative note compliance rose from 82% to 100%.

The brief operative note was established for clinical use in July of 2012, and survey data was collected between October 2012 and May 2013. During the data collection period, a total of 1,936 neurosurgical cases were performed. In October 2012, the percentage of brief operative notes completed within 30 minutes was 82%. Four months later, compliance for completion of the brief operative note within 30 minutes was 98%. At the end of the study period, the compliance rose to 100%, as evident in Figure 1. We believe that this increase over time was due to heightened buy-in by the residents and nurses to participate in this protocol because of its ability to lead to smoother transitions of care.

A total of 29 survey responses (15 RN, 14 resident) were analyzed. The elements of a brief operative note most important to ICU RNs and residents were the exact procedure performed (ICU RNs - 87% either critical or important; residents - 91% either critical or important), fluids administered (ICU RNs - 100% either critical or important; residents - 50% either critical or important), and estimated blood loss (ICU RNs - 94% either critical or important; residents - 85% either critical or important). Additionally, a majority of residents (93%) felt that communicating the postoperative disposition in the immediate postoperative period, within four hours, was critical (43%) or important (50%). 

As mentioned earlier, 63% of residents surveyed in a previous study reported a lack of instruction towards what constitutes an effective handoff; in this study, when asked whether this lack of training leads to a lack of documentation which ultimately adversely affects postoperative patient care, a large majority of residents agreed (Always - 7%, Often - 14%, Sometimes - 57%).

One hundred percent of residents either strongly agreed (67%) or agreed (33%) that brief operative notes improve safety of postoperative patients in a neurosurgical setting. Overall, survey data indicated that a large majority of both ICU RN (Strongly Agree - 64%, Agree - 29%, total = 93%) and resident (Strongly Agree - 55%, Agree - 45%, total = 100%) respondents felt that brief operative notes provided a useful source of information in their clinical practice.

## Discussion

In 2009, the Joint Commission established guidelines for handoff communication between healthcare providers. It was requested that all accreditation programs implement structured transfer of care communications among healthcare professionals that include in-person encounters [12]. The Joint Commission analyzed the cause of sentinel events from 2012 to 2014, and one of the foremost causes was communication errors among physicians and nurses during transitions of patient care [13]. 

Among other data, the guidelines recommend that in surgical settings the transfer of information should include procedure performed, procedure complications, if applicable, anesthesia and analgesia type, intraoperative medications and allergies, estimated blood loss and fluid status, and post-surgical plans. These guidelines were developed in part based on successful strategies utilized in other high-risk environments, including nuclear power plants and mission control centers of the National Aeronautics Space Administration (NASA) [14]. During 16 observed shift handoffs at the NASA center, 8/75 questions were repetitive in nature and used to detect errors and confirm understanding, such as “Do you know that for sure?” A key component of the NASA handoff communication is verification and repetition of information by the recipient. Our handoff system, composed of both electronic documentation and a face-to-face encounter between resident and nurse, also achieves this goal of verification and repetition of critical information [5, 9-10]. The invaluable nature of reinforced communication should not be undermined when seeking robust handoff implementation.

Wide variation exists in the transfer of patient care among healthcare professionals. For instance, face-to-face communication, electronic documentation, or communication via phone can all be utilized to relay critical information. Solet, et al. compared these mediums of communication in patient handoffs in the medicine ward of a large teaching hospital. Upon analyzing mediated and non-mediated forms of handoffs, the authors argued that direct face-to-face communication has the greatest potential for reducing medical error [11]. As compared to electronic documentation or transfer of information via telephone, in-person verbal contact entails cues, such as facial expression, posture, and eye contact, may provide additional information regarding the level of concern about the patient’s medical problems while ultimately enhancing the receiver’s ability to interpret the information being relayed. The argument is also made that electronic documentation may often leave ambiguities and give rise to unanswered questions, which may adversely affect patient care. It is suggested that the optimal handoff among healthcare professionals regarding hospitalized patients should involve a combination of both verbal and electronic communication. A similar study performed in an emergency room setting identified “high-risk” patients similar to patients in the ICU, in which the potential for a breakdown in communication is costly, and analyzed handoff strategies between emergency medicine residents and hospital medicine clinicians. It was concluded that standardized tools in the ED are not commonly used, but that ideally face-to-face communication would be best in order to maximize patient safety [15].

Ample evidence also exists to indicate that standardized transfer of care procedures is more efficacious in limiting medical errors than are non-standardized handoffs. An example of this is a multicenter study that trained residents in pediatric residency programs about Illness Severity, Patient Summary, Action Items, Situational Awareness and Planning, and Synthesis by Receiver (I-PASS), a mnemonic-based handoff protocol that offered a predictable structure to enhance information transfer among healthcare professionals. Across nine participating hospitals over a six-month intervention period and over a total of 10,740 patient admissions, this system significantly increased patient safety by reducing medical errors and the rate of preventable adverse effects by 23% and 30%, respectively [16]. Paired with computerized handoff tools, this system ultimately enabled a reduction in medical errors by collaborative crosschecks among peers. It is important to note, however, that little evidence exists that standardized handoff mnemonics in and of itself provides measurable benefits [17]. Although this bundle was focused primarily on inpatient pediatric care, the principles of standardized handoffs are invaluable in any situation with potential for a breakdown in communication, especially in the neurosurgical environment. We believe that our resident-led implementation of structured patient care transfer is solidified not only through its standardized electronic checklist approach but also by face-to-face communication.

In addition to exhibiting interactivity, handoffs should be conducted with limited interruptions in order to enhance communication. However, the complete minimization of distractions is not always feasible, especially in the bustling neurosurgical environment. It is important to note that our study focused on neurosurgical perioperative interdisciplinary handoffs, namely among residents and registered nurses, involving patients who require high acuity of care postoperatively. Since there are different levels of training among these interdisciplinary healthcare professionals, this provides another reason why strategic and comprehensive transfers of care are vital to proper patient care. In this paper, we have discussed our two-stage system that utilizes both written and verbal communication to focus the transfer of critical perioperative data, with the use of reiterations to minimize inaccurate data communication among healthcare professionals.

Our results demonstrate the feasibility and adoptability of such an approach at an academic training environment; yet, there are several limitations to the study. First, because some elements of the brief operative note existed in EPIC prior to our study and were used only intermittently, we lack clear pre-intervention data. Second, due to the presence of patients from other surgical subspecialties in the postoperative recovery area, survey results were not obtained from post-anesthesia care unit (PACU) nurses. Lastly, other patient safety initiatives were also launched concurrently with the standard handoff initiative, including a perioperative safety video [18]. The video was required material for all healthcare providers of neurological care to patients in the preoperative setting, including 155 PACU and ICU RNs. Although not specifying a structured format, the video encouraged face-to-face handoff of the patient postoperatively between members of the surgical and nursing team at the destination unit. A questionnaire administered to RNs regarding attitudes on handoff effectiveness contained the following statement: “Important patient care information is often lost when transferring patients from one unit to another.” In that survey, the percentage of respondents who either disagreed or strongly disagreed to this statement increased from 52% before the video (July 2012) to 61% after the video (July 2013).

This survey thus overlapped with our resident-led postoperative note and handoff system, making it difficult to disambiguate the effects of the interventions. In addition to these interventions, a University of California-wide initiative (UC Care Check) was developed in March 2014 with the aim of improving neurosurgical patient outcomes and care experiences across all five neurosurgical departments in the UC health care system. A comprehensive and quantitative analysis of each of these interventions is currently being planned.

## Conclusions

We report a standardized handoff system, which includes both written documentation and face-to-face communication, to facilitate the transfer of neurosurgical patients to the postoperative setting among interdisciplinary healthcare professionals, namely, residents and registered nurses. Our results indicate the importance of accurate and timely transfer of critical postoperative data to RNs as a means of maximizing patient safety and care continuity, in particular with face-to-face contact being invaluable in the surgical environment as a means of reinforcement. More efforts are needed to establish comprehensive, standardized surgical handoff neurosurgical strategies consisting of verbal and written documentation. Future analyses are ongoing to quantify the impact of combined quality improvement initiatives in our healthcare system on the outcomes of neurosurgical patients.
